# The Antivivisection Memorial

**Published:** 1891-11-14

**Authors:** 


### The Hospital

A Weekly Journal of

Science, Medicine, Nursing, and Philanthropy.

*For Hospitals, Asylums, Infirmaries, Dispensaries, Medical Practitioners, Students, Nurses, and the Charitable Public.*

Vol. XI.—No. 268.] [Nov. 14, 1891.

### The Antivivisection Memorial.

An army of 41,315 volunteers with such leaders as Lord Tennyson, Cardinal Manning, Mr. Ruskin, Dr. James Martineau, Mr. Froude, Mr. Luther Holden, F.R.C.S., late President of the Royal College of Surgeons of England, the Lord Chief Justice, four Dukes, fourteen Bishops, a large number of Duchesses and Peeresses, and fifty medical men, constitutes a unique sight, and is worthy of some consideration. Those volunteers are banded together for the total suppression of vivisection; and, as a first step to that consummation, for the prevention of the issue of a State License to the proposed Institute of Preventive Medicine. There is thus a conflict established between opposing elements in the progress of civilisation. The issue of the conflict will be ultimately decided, like every other great question in this country, by public opinion. The English people, on the whole, desire to do right. Of course, they also wish to wring from Nature every secret which may be of use to them, and to make all natural things, as far as possible, their servants rather than their masters.

It is a striking circumstance that in one of the earliest historical records we have there should be given an account of a wide-reaching general command, issued to man, which bears on this very subject. According to the record, man was commanded to increase and multiply, and also to replenish the earth and subdue it. He was, so says the record, divinely bidden to have dominion over the fish of the sea, the fowl of the air, and all living creatures that move on the face of the earth. From that early time to the present men have not only claimed, but exercised dominion and proprietorship over all those creatures which we call lower animals. All the races of men have exercised the same rights in a greater or less degree. They have deprived the animals of freedom; they have mutilated them to make them the better subserve human purposes; they have made them beasts of burden; they have killed them for food. They have never hesitated to take them in traps, to slaughter them with rude weapons, to poison them, to shoot them, to use them, in fact, in any and every way which might be most conducive to human convenience, pleasure, or profit. All these are the common-places of world-wide experience,

The modern world, in England at any rate, now finds itself divided into two camps about this question of animal subjection. It has found itself in two camps before, on such subjects as slavery, for example, and the treatment of undeveloped and comparatively defenceless tribes and peoples. The opposing camps represent two distinct, but not necessarily hostile lines of thought. The one stands for pity, consideration for creatures that are at the mercy of man, the doing by men as they would be done by if they, by some stroke of destiny, could be made to change places with the lower animals. The other stands for knowledge, for the right to know in order to gain the power to do, for the sacrificing of the lower creatures in order to benefit the higher. It is an old conflict. The pitiful ones represent a noble class of ideas, associated with great and venerable names. The knowledge seekers also represent an honoured class, and they, too, fight under the ægis of names admired and renowned in the many-coloured story of the world.

It is a legitimate, nay, a worthy ambition, to ardently desire knowledge on the one hand, and it is noble on the other to wish to do to the lower animals as we should wish them to do to us if we were the lower animals and they were men. The conflict is painful to at least one mind; it must, indeed, be painful to many. Consider the position of the humane medical man. His working life is spent at the bedside of the sick. He witnesses, constantly, pains that amount to agony: pains that prevent sleep, that drive the sufferer to distraction, and almost to madness; pains and diseases that kill. He is often compelled to watch a strong man dying in the midst of his years: dying and leaving his wife and helpless children destitute. He has to look upon suffering children, poor little creatures that have not reached the age of thought, or acquired by experience the power of endurance; he is compelled to see them desperate and beside themselves with pain and hopeless disease. Their eyes and their struggles appeal from mother to father, from father to nurse, and from nurse to doctor for help and health which, very frequently, not one of them has the power to give. What does the physician say to himself when he is called upon to take the guiding part in scenes like these? If I had but knowledge, he says; if I had but power; if heaven, earth, or the sea would but yield up to my search something whereby this anguish might be relieved, this life saved, I could live my life and perform my painful tasks content and satisfied. Is it not evident that the nobler the doctor's mind, the more he must desire knowledge and power for the proper doing of his responsible and oftentimes most distressing work?

The antivivisectionists must try to put themselves in the doctor's place. Many a medical man of the present day is in a truly unhappy frame of mind on the subject of vivisection. Sir Joseph Lister is one of the most humane and also one of the most sensitive of men. He is a type of thousands of British physicians and surgeons. Such men stand in the fore-front of the world's development and advance. They are there by the right of proved capacity. They have but one purpose, and that is to advance on the lines of truth and righteousness. They deserve the sympathy of all men of candour and right feeling. Mr. Ruskin ought to sympathise with them, and so also ought Cardinal Manning, the Lord Chief Justice, and all the other noble-minded people whose pity for the animals that are at the mercy of man is so great. What can such men as Sir Joseph Lister do? They feel assured that a certain amount of valuable knowledge is to be gained by vivisection; and a certain more or less considerable increase of power over pain, disease, and death. These are the possibilities that lie before them. May they not, with some show of reason, contend that it is a divine hand that is beckoning them forward in the direction of more light and of greater control over such dread enemies as pain, disease, and death? May they not, in looking back over the history of the world, contend that the divine providence has permitted all manner of dreadful deeds to be done, and every variety of exquisite agony to be endured, and out of the chaos of apparently purposeless anguish has brought forth clearer light and nobler and more beautiful life?

But if we ask Mr. Ruskin and Cardinal Manning to put themselves in the place of Sir Joseph Lister and those who stand by his side, must we not also ask Sir Joseph Lister and other pioneers of science to put themselves in the place of Mr. Ruskin and those who sympathise with him? It is a dread act for a man with his man's intellect to take a living and sentient animal, with its less than child's intelligence, to bind it fast, and to cut slowly into its living frame in search of knowledge which may often be of small import, and sometimes of no import at all. The thing can only be done under an overpowering sense of responsibility and necessity. The doer of it must put himself in the place of God. He is the highest power known to the lower animals: their God, in fact. He himself is a lower animal to some higher being or beings He would look with a pathetic appeal to any higher being who should propose on any particular day to do to him as he does to the lower animals under his control. One can imagine the higher being yielding to the gentle influences of pity, and consenting to allow him to escape, even at the cost of some loss of valuable knowledge. Such feelings as are here imagined undoubtedly animate the minds of Mr. Ruskin, Cardinal Manning, Mr. Luther Holden, and others. They are entirely worthy and honourable feelings; nay, they are among the most noble feelings of humanity. Such feelings have been chief instruments in raising humanity to its present level; they will continue to be chief instruments in its further and higher development.

It may be asked, What is the use of an article of this uncertain tone, which seems to take neither one side nor the other? But it may also be asked, Has the time come for taking sides? Is it not rather the time for a deeper consideration of the whole question? Would it not be well that representatives of both sides should meet and talk with each other face to face on the subject? The question is one of the most important in the whole region of morals and human advancement. If scientific progress seems to one party to be at stake, the noblest humanity seems to the other to be trembling in the balance. The writer, as a medical man, feels that vivisection is eminently a question for the good and honourable men of both sides to consider afresh with patience, intelligence, and candour.

